# *Ab initio* phasing by molecular averaging in real space with new criteria: application to structure determination of a betanodavirus

**DOI:** 10.1107/S2059798316007695

**Published:** 2016-06-23

**Authors:** Masato Yoshimura, Nai-Chi Chen, Hong-Hsiang Guan, Phimonphan Chuankhayan, Chien-Chih Lin, Atsushi Nakagawa, Chun-Jung Chen

**Affiliations:** a Life Science Group, Scientific Research Division, National Synchrotron Radiation Research Center, 101 Hsin-Ann Road, Hsinchu 30076, Taiwan; b Institute of Biotechnology and University Center for Bioscience and Biotechnology, National Cheng Kung University, Tainan 701, Taiwan; c Institute for Protein Research, Osaka University, 3-2 Yamadaoka, Suita, Osaka 565-0871, Japan; d Department of Physics, National Tsing Hua University, Hsinchu 39943, Taiwan

**Keywords:** *ab initio* phasing, NCS averaging, cross-crystal averaging, multi-crystal averaging, phasing power, icosahedral virus structure, free fraction

## Abstract

Molecular averaging, including noncrystallographic symmetry (NCS) averaging, is a powerful method for *ab initio* phase determination and phase improvement. Applications of the cross-crystal averaging (CCA) method have been shown to be effective for phase improvement after initial phasing by molecular replacement, isomorphous replacement, anomalous dispersion or combinations of these methods. Here, a two-step process for phase determination in the X-ray structural analysis of a new coat protein from a betanodavirus, *Grouper nervous necrosis virus*, is described in detail. The first step is *ab initio* structure determination of the *T* = 3 icosahedral virus-like particle using NCS averaging (NCSA). The second step involves structure determination of the protrusion domain of the viral molecule using cross-crystal averaging. In this method, molecular averaging and solvent flattening constrain the electron density in real space. To quantify these constraints, a new, simple and general indicator, free fraction (ff), is introduced, where ff is defined as the ratio of the volume of the electron density that is freely changed to the total volume of the crystal unit cell. This indicator is useful and effective to evaluate the strengths of both NCSA and CCA. Under the condition that a mask (envelope) covers the target molecule well, an ff value of less than 0.1, as a new rule of thumb, gives sufficient phasing power for the successful construction of new structures.

## Abstract 2

A two-step process of phase determination in the X-ray structural analysis of the coat protein of a betanodavirus is described. A new indicator, the free fraction, for molecular averaging in real space is introduced to effectively evaluate the phasing power in order to enhance the success of determining new structures.

## 1. Introduction   

The molecular averaging method in real space, including noncrystallographic symmetry (NCS) averaging and cross-crystal averaging (CCA), coupled with solvent flattening, has proved to be powerful in improving the determination of the phases in protein crystallography. As a case in point, NCS averaging (NCSA) with phase extension of icosahedral viruses is a common procedure for phase improvement after initial calculations based on molecular replacement (MR) using a density map from a cryo-electron microscope, a similar model or initial experimental phases from isomorphous replacement or anomalous dispersion. Since the successful structure determination of the human common cold virus serotype 14 (Rossmann *et al.*, 1985 ▸; Arnold *et al.*, 1987 ▸), several virus structures have been solved using NCSA with phase extension. As the phasing power of NCSA is effective, the possibility of *ab initio* phase determination has been proposed. The feasibility of *ab initio* phase determination has been demonstrated for spherical viruses (Tsao *et al.*, 1992 ▸). *Ab initio* trials and procedures have been reported for known icosahedral viruses (Taka *et al.*, 2005 ▸). An assessment of the application of NCSA to icosahedral viruses, together with a detailed investigation of successful and unsuccessful cases, has also appeared (Plevka *et al.*, 2011 ▸).

When the initial phases were unsatisfactory, the CCA method has been utilized for phase improvement (Taylor, 2010 ▸). Many cases were improved with CCA from initial phasing of various types: MR (Isupov *et al.*, 2004 ▸), multiple isomorphous replacement (MIR; Crennell *et al.*, 2000 ▸; Chen *et al.*, 2005 ▸) and single-wavelength anomalous diffraction (SAD; Su *et al.*, 2010 ▸). The phasing power of CCA for the improvement of initial phases from MR to obtain a partial unknown structure has been demonstrated and discussed (Li & Li, 2011 ▸), but there has been no attempt to achieve *ab initio* phasing by CCA.

*Nodaviridae* are a family of positive-sense single-stranded RNA viruses with a non-enveloped *T* = 3 icosahedral capsid. *Alphanodavirus* and *Betanodavirus* are the two major genera. Several structures of alphanodaviruses, including *Nodamura virus* (NoV; PDB entry 1nov; Zlotnick *et al.*, 1997 ▸), *Pariacoto virus* (PaV; PDB entry 1f8v; Tang *et al.*, 2001 ▸), *Black beetle virus* (BBV; PDB entry 2bbv; Wery *et al.*, 1994 ▸) and *Flock house virus* (FHV; PDB entry 4fsj; J. A. Speir, Z. Chen, V. S. Reddy & J. E. Johnson, unpublished work), have been solved. Various strains of the genus *Betanodavirus* have been isolated from several fishes, such as *Striped jack nervous necrosis virus* (SJNNV), *Tiger puffer nervous virus* (TPNNV), *Red-spotted grouper nervous necrosis virus* (RGNNV) and *Barfin flounder nervous necrosis virus* (BFNNV) (Nishizawa *et al.*, 1997 ▸). These betanodaviruses have a similar viral genome and cause viral nervous necrosis (VNN). We have recently determined the first crystal structures of a betanodavirus, *Grouper nervous necrosis virus* (GNNV), in various forms: a complete *T* = 3 GNNV-like particle (GNNV-LP) at 3.6 Å resolution, a *T* = 1 icosahedral subviral particle of the truncated surface domain (S-domain) at 3.1 Å resolution and the individual protrusion domain (P-domain) of the GNNV coat protein (CP) at 1.2 Å resolution (Chen *et al.*, 2015 ▸). In this paper, we describe the processes used in phase determination of the *T* = 3 GNNV-LP and the P-domain in detail.

To determine the new structure of GNNV-LP, we applied molecular-averaging methods in two steps. Firstly, an NCSA *ab initio* phasing trial with a similar envelope from a different virus was performed. The structure of the whole virus-like particle (VLP) of *T* = 3 GNNV was successfully obtained. Atomic modelling with the amino-acid sequence of the S-domain of the *T =* 3 GNNV capsid protein was straightforward. The density map of the P-domain region in the *T* = 3 GNNV-LP was, however, insufficient for complete model building, so that only partial main chains could be traced. An analysis of the *T* = 3 GNNV particle structure led us to design a truncated form consisting of only the P-domain of the CP. Several crystal forms of the individual P-domain were subsequently obtained at high resolution (1.8 Å for phasing, 1.2 Å for final refinement). Secondly, utilizing the cut-out envelope of the P-domain region of the *T* = 3 GNNV-LP crystal, we applied CCA to the various crystal forms of the P-domain to acquire the correct phases, including the phases for the high-resolution data. In this manner, the structure of the individual P-domain was successfully built with good quality and was subsequently used for complete model building and refinement of *T* = 3 GNNV-LP.

Following earlier work (Arnold & Rossmann, 1986 ▸), a new and simpler quantification of the power of molecular averaging is introduced in our work. In molecular averaging, some portions of the electron density in the unit cell of the crystal are constrained by both molecular averaging and solvent flattening. We quantify an indicator, named the free fraction (ff), which corresponds to the ratio of the constrained volume and the unconstrained volume and can be easily applied to the case of CCA. Here, we discuss our attempts in applying ff to phase determinations with both NCSA and CCA.

## 2. Methods and results for phase determination of icosahedral *T* = 3 GNNV-LP using NCS averaging   

### 2.1. Data collection and processing   

Crystals of *T* = 3 GNNV-LP were first screened on beamlines BL13B1, BL13C1 and BL15A1 at NSRRC, Hsinchu, Taiwan and BL12B2 at SPring-8, Harima, Japan; the complete X-ray diffraction data sets were collected on BL44XU at SPring-8 with a charge-coupled device (CCD) detector (MX225-HE, Rayonix). The crystals of GNNV-LP belonged to the monoclinic space group *C*2, with unit-cell parameters *a* = 477, *b* = 422, *c* = 337 Å, β = 134°. Diffraction images were collected with an oscillation angle of 0.3° per frame and an exposure duration of 3 s at a wavelength of 0.9 Å. The high-resolution data set (50–3.6 Å) was collected first.

To assist *ab initio* phase determination, data at very low resolution are essential. During data collection, the beamstop was moved to the downstream side at 95 mm from the crystal, as near to the detector as possible; an X-ray beam of wavelength 1.2 Å was used to include the lowest resolution spots (266–12 Å). The images at this low resolution were collected with an oscillation angle of 0.5° per frame and an exposure duration of 3 s. The entire data sets, including those at very low and high resolutions, were recorded with an X-ray beam size of 50 µm on separate diffraction regions of a long GNNV-LP crystal of dimensions 0.1 × 0.1 × 0.3 mm.

For data processing, including integration and scaling, we used *HKL*-2000 (Otwinowski & Minor, 1997 ▸). During the processing of the data at the lowest resolution, the rejection criterion to select diffraction spots was changed to the loosest criterion in order to prevent the rejection of any spots. Even though heavily overloaded spots were observed in the region of very low resolution, we collected these spots without rejections; the number of missing reflections at the lower resolution side was thus kept to a minimum. Only two reflections, (*h* *k* *l*) = (3 1 −1) and (3 1 −2), were missed in the resolution range below 100 Å. A total of 600 frames were taken for the high-resolution data set and 360 frames for the low-resolution data set; scaling was performed concurrently with these 960 frames in total. The data set at the highest resolution eventually contained 499 184 reflections from 50 to 3.6 Å with a completeness of 98% for use in refinement.

### 2.2. Phase determination of *T* = 3 GNNV-LP   

Self-rotation functions were calculated to determine the orientations of the icosahedral symmetries (twofold, threefold and fivefold axes) using *MOLREP* (Vagin & Teplyakov, 2010 ▸) from the *CCP*4 program suite (Winn *et al.*, 2011 ▸). The orientation of the GNNV-LP particle was suggested to be the rotated orientation with *CCP*4 polar angles (ω, φ, κ) = (90, 90, 40°) from the icosahedral convention of orthogonal coordinate axes and fold axes: 2(*z*)-5-3-(*x*)2 (Arnold *et al.*, 1984 ▸). There were two GNNV-LP particles in the monoclinic *C*2 unit cell, with one twofold NCS axis of the virus icosahedral particle coinciding with the crystallographic twofold axis. One asymmetric unit contained half of a GNNV-LP particle or 30 copies of the icosahedral symmetric unit. 60 matrices for icosahedral symmetries were thus generated, of which 30 were used in the averaging calculations as the initial matrix operators for NCSA. To simplify the matrix parameters, we arranged the centre of one GNNV-LP icosahedral particle to be located at the origin (0, 0, 0).

**Figure 1:**
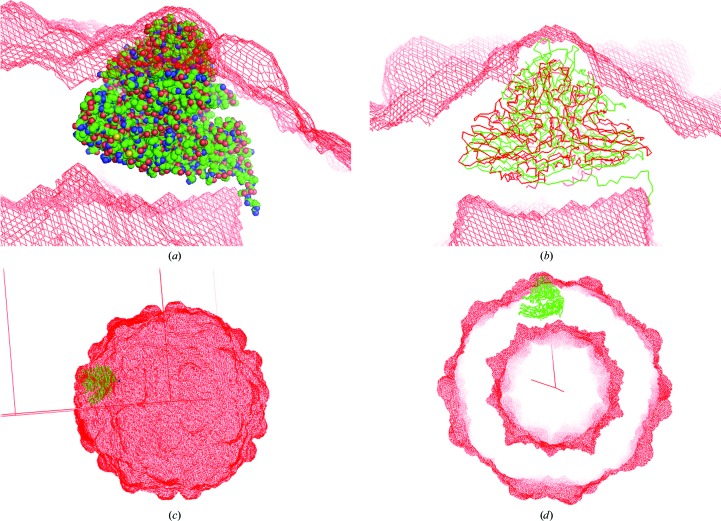
The initial mask created from the atomic structure of *T* = 3 FHV with a large mask radius of 13 Å around each atom. (*a*) The initial mask corresponding to the finally obtained icosahedral asymmetric unit (i-ASU) of *T* = 3 GNNV-LP. (*b*) The initial mask with C^α^ tracings of the i-ASU of *T* = 3 GNNV-LP (green) and the i-ASU of *T* = 3 FHV (red). (*c*) Entire presentation of the initial mask with C^α^ tracing of the i-ASU of *T* = 3 GNNV-LP. (*d*) A cross-section view of the entire initial mask with *T =* 3 GNNV-LP.

For the *ab initio* phasing method, which used NCSA with phase extension, an appropriate mask was necessary to separate two regions: the protein region to be NCS-averaged and the solvent region to be flattened. The initial mask for NCSA and solvent flattening was created from the atomic structure of *T =* 3 FHV (PDB entry 4fsj), a disparate structure, with a larger mask radius of 11–13 Å around each atom (Fig. 1 ▸). The mask ranges with those protein regions overlapping with the neighbouring particle were truncated at half the distances of the nearest atoms. As the starting model, a spherical-shell model with uniform density was used; the outer and inner radii of the model were 159 and 119 Å, respectively. These radius values were typical initial parameters for the *T* = 3 virus particles suggested in previous work (Taka *et al.*, 2005 ▸). A comparison of the data with the scattering amplitudes of the spherical-shell model is shown in Supplementary Fig. S1. The model reproduces similar nodes and peaks to the data of lower resolution (>60 Å).

**Figure 2:**
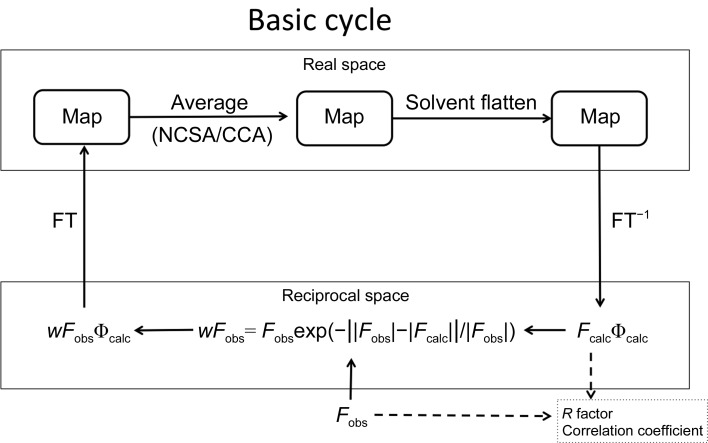
A schematic diagram of the basic iteration cycle of the averaging method (top) and a diagram showing the procedure around CCA phasing of the P-domain.

Using data with the ‘initial’ parameters of the above-described initial mask, the initial spherical model and the initial NCSA matrices, *ab initio* phasing with NCSA was subsequently applied. A basic NCSA cycle involved iterative calculations between real space and reciprocal space, which were linked with the Fourier transform (FT) and inverse FT (FT^−1^). In this cycle, NCSA and solvent flattening were applied to the density map in real space. A phase combination of Φ_calc_ with *w* × *F*_obs_ by Rayment weighting (Rayment, 1983 ▸) was applied in reciprocal space, in which *w* corresponds to
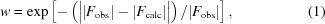
where *F*_obs_ is the observed structure factor and *F*_calc_ is the calculated structure factor from the processed density map. A schematic diagram to explain the basic cycle is presented in Fig. 2 ▸. In the cycling calculation, we used *AVE* from *RAVE* (Kleywegt & Jones, 1999 ▸) for NCSA and solvent flattening, *FFT* and *SFALL* (*CCP*4; Winn *et al.*, 2011 ▸) for FT and FT^−1^, and *SFTOOLS* (coded by B. Hazes) to calculate the weighting factor. *RSTATS* (coded by S. E. V. Phillips) was used to scale *F*_calc_ to *F*_obs_ and to calculate *R* factors and correlation coefficients between *F*_obs_ and *F*_calc_.

**Figure 3:**
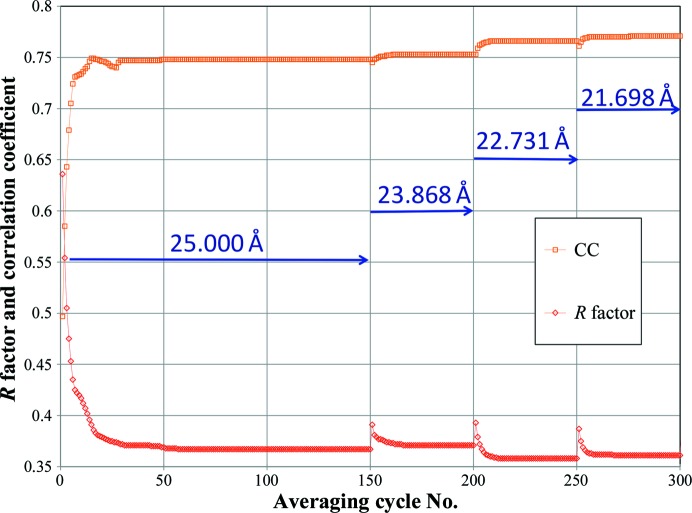
The correlation coefficient (CC) and *R* factor comparing *F*_obs_ and *F*_calc_ during convergence of the cycles in the first low-resolution step and the following three phase-extension steps of the first successful *ab initio* phasing with the initial mask and the initial transfer matrices.

**Figure 4:**
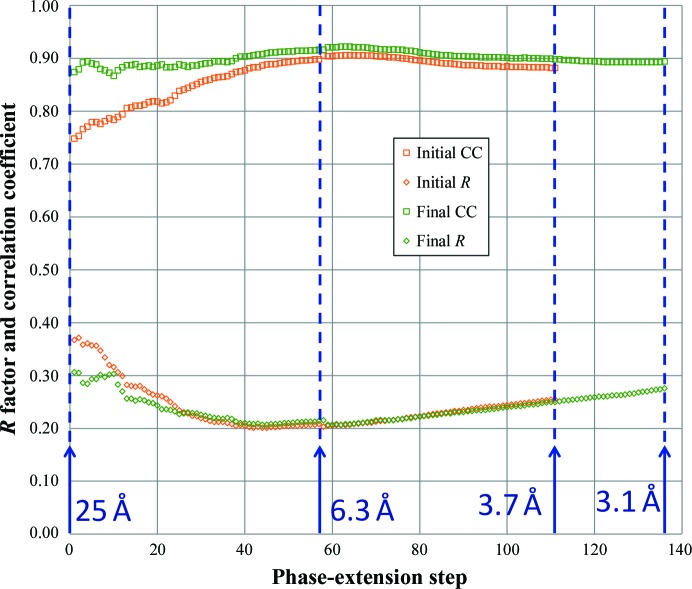
The correlation coefficient (CC) and *R* factor comparing *F*_obs_ and *F*_calc_ after convergence in each phase-extension cycle. The blue arrows and digits show the maximum resolution in each cycle. ‘Initial’ means the first successful *ab initio* phasing with the initial mask and the initial transfer matrices. ‘Final’ means phase extension with the final optimized mask and transfer matrices.

**Figure 5:**
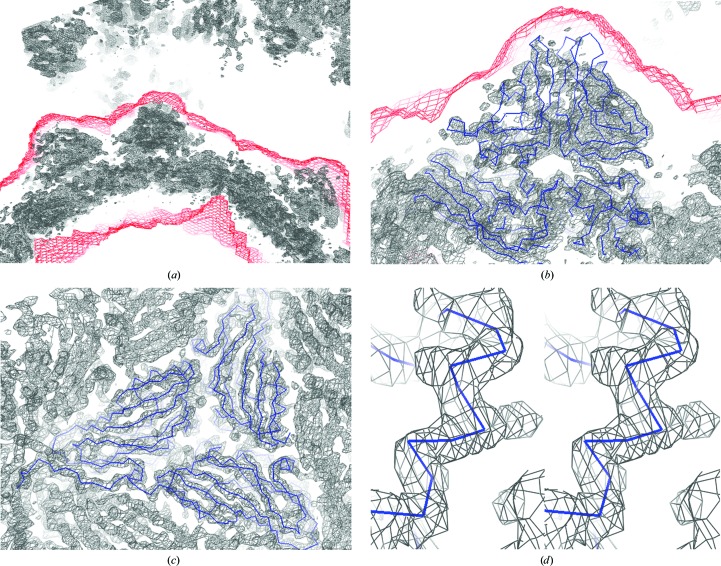
Electron-density maps obtained *ab initio* at a resolution of 3.7 Å using the initial mask and the initial transfer matrices. The red meshes show the initial mask used; the blue lines show the backbones of the final refined model. (*a*) A cross-section view of the map for the *T* = 3 GNNV particle with the initial mask. The map was contoured at 1.5σ. (*b*) An enlarged view of the map for the S-domain and P-domain with the initial mask and the final model. (*c*) A view from the inside of the particle. An electron-density map for the S-domain parts can be clearly recognized. (*d*) A stereo map at 1.0σ for helices in the S-­domain.

After more than 100 cycles of iteration at 25 Å resolution, phase extension was performed from 25 Å to the highest resolution (initially 3.7 Å to verify phasing and extended to 3.1 Å while testing the map quality) with 50 iterations in one reciprocal-lattice step (∼1/*a*; *a* is the unit-cell dimension). This process is referred to as ‘the procedure’ hereafter. The numbers of both iterations (>100 cycles) at low resolution and phase-extension steps (>50 cycles) sufficed for convergence (Fig. 3 ▸). We performed 111 steps in phase extension from 25 to 3.7 Å (Fig. 4 ▸). An interpretable electron-density map was successfully obtained with the procedure using the initial parameters (Fig. 5 ▸).

The electron density of helical structural elements such as α-helices in GNNV-LP at 3.7 Å resolution (or better) was used to examine the correct enantiomorphism of the phase sets (Fig. 5 ▸). To improve the electron density, we updated the mask based on the resulting map after every procedure. Each procedure was initiated from the spherical-shell uniform density model with the updated mask and the NCS operators. These NCS operators were refined as the icosahedral orientations by selecting the highest correlation coefficient among many steps with varied κ angles (for example from 39.0 to 41.0° with a 0.1° step in the ‘initial procedure’). After around ten iterations of the procedure, the correlation coefficient converged to give the highest value; we refer to this last procedure as the ‘final procedure’. The final icosahedral orientation was determined as (ω, φ, κ) = (90, 90, 40.025°), in which steps of 0.002° were used to determine the final κ value. The best values of the *R* factor and the correlation coefficient appeared to be 0.20 and 0.92, respectively, at ∼6 Å resolution. The overall values of these calculations are given in Table 1 ▸; the progress of phase extension is shown in Fig. 4 ▸. All graphics showing molecular structures were produced with *PyMOL* (http://www.pymol.org/).

### 2.3. Phase improvement and model building   

In the last cycle of phase improvement, *DM* (Cowtan, 1994 ▸) was used for NCSA with refinement of the NCS operators. The quality of the final electron-density map was sufficient to build an atomic model apart from the P-domain region of *T* = 3 GNNV-LP. We initially suspected that the P-domain of *T* = 3 GNNV-LP failed to conform to the strict icosahedral symmetry, but utilizing *DM* with various trials, including the individual mask and the NCS operator around the P-domain, did not significantly improve the density map of the P-domain. From the density map of *T* = 3 GNNV-LP, the linker region, which links the surface and protrusion domains, was identified. According to this useful information, the corresponding sequence and the spatial arrangement of the individual protrusion domain region were readily obtained for further analysis, which is described in the following section. Model building was performed manually with *Coot* (Emsley *et al.*, 2010 ▸).

## 3. Methods and results for phasing of the protrusion domain by cross-crystal averaging   

### 3.1. Crystallization   

From the information of the corresponding sequence of the P-domain of *T* = 3 GNNV and a sequence alignment of various betanodaviruses, we constructed the truncated P-domain and performed crystallization experiments. The crystallization procedure of the individual P-domain was conducted using approaches similar to those used for GNNV-LP. We obtained several crystallization conditions that produced crystals in various space groups, including *C*2, *P*2_1_2_1_2_1_ and *P*3, using 0.8 *M* lithium chloride, 0.1 *M* Tris–HCl pH 8.5, 32%(*w*/*v*) PEG 4000 (*C*2), 0.2 *M* KCl, 0.1 *M* magnesium acetate, 0.05 *M* sodium cacodylate pH 6.5, 10%(*w*/*v*) PEG 8000 (*P*2_1_2_1_2_1_) and 0.2 *M* NaCl, 0.1 *M* Tris–HCl pH 8.0, 20%(*w*/*v*) PEG 4000 (*P*3). All crystals appeared within one week. Single crystals (∼0.1 × 0.1 × 0.1 mm) were transferred to the respective crystallization solution containing glycerol (20%) as a cryoprotectant and were flash-cooled with liquid N_2_ for data collection.

### 3.2. Data collection and processing   

X-ray diffraction data were collected from the P-domain crystals with an oscillation angle of 1.0° per frame and an exposure duration of 20 s at a wavelength of 1.0 Å on beamline BL13C1 at NSRRC equipped with a CCD detector (Q315r, ADSC). All data sets were processed with *HKL*-2000 (Otwinowski & Minor, 1997 ▸). The statistics of data collection for four different crystal forms are presented in Table 1 ▸. The crystal forms are named P212121, C2D, C2S and P3, respectively, in terms of the corresponding space group and the number of NCS: S (single) or D (double). For the monoclinic space group *C*2, we obtained two crystal forms with large and small unit cells (Table 1 ▸). From analyses of the Matthews coefficient (Matthews, 1968 ▸), C2D was expected to have two molecules in the asymmetric unit, whereas C2S contained one.

### 3.3. Phase determination using cross-crystal averaging   

**Figure 6:**
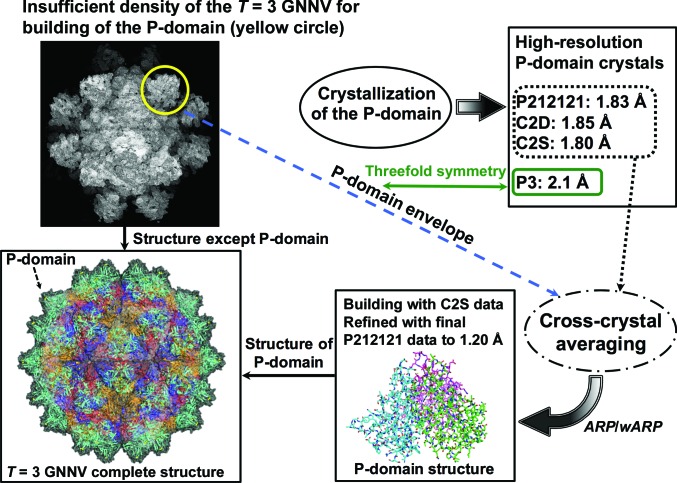
A flow chart showing the contribution of each crystal form of the P-domain to phasing calculations.

An initial attempt to determine the structure of the P-domain using the MR method with the previous partially traced model of the P-domain from GNNV-LP was not straightforward and failed. We then performed the CCA method using the three data sets P212121, C2D and C2S. A flow chart showing the contribution of each crystal form of the P-domain to phasing calculations is shown in Fig. 6 ▸.

**Figure 7:**
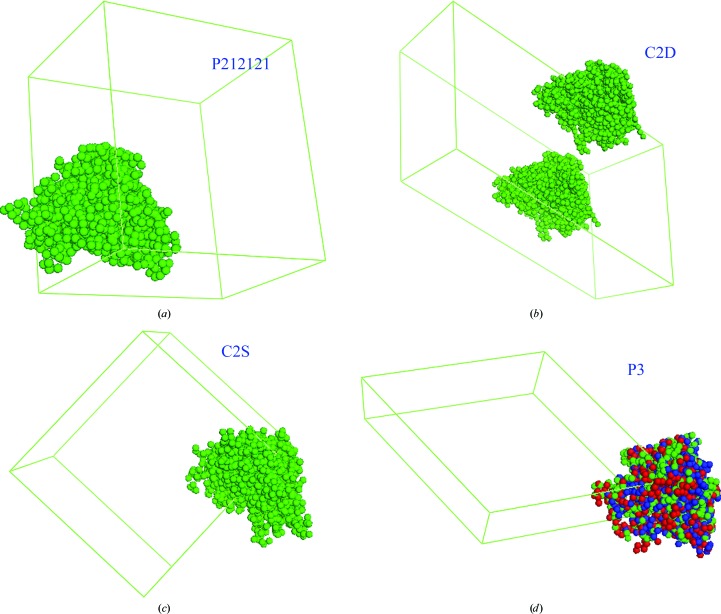
MR solutions of the P-domain envelope from the *T* = 3 GNNV electron density for each crystal form: (*a*) P212121, (*b*) C2D, (*c*) C2S, (*d*) P3. The P-­domain envelope consists of dummy atoms (green spheres). In (*d*) the other two crystallographic symmetry solutions are also presented and are coloured red and blue. The envelope form was expected to be that of a trimer.

From the electron-density map of *T* = 3 GNNV-LP, the coordinates of the (regular interval) grid points of the map in the region of the P-domain with density greater than 0.4σ were selected. On these grid coordinates (not on density peaks), dummy C atoms were placed to form an envelope of the P-domain as a PDB-format file. This P-domain envelope served as the initial MR model for each crystal. Using *Phaser* (McCoy *et al.*, 2007 ▸), we found the MR solutions with the highest scores. Fig. 7 ▸ shows the initial models with dummy atoms in each of the crystal forms.

From these MR solutions, the translocation matrices for CCA were derived. We used four translocation matrices: one identity matrix from the molecule of P212121 to itself, two matrices to the two molecules of C2D and one matrix to the one molecule of C2S. The mask is necessary to define the region of molecules as in the NCSA case; it was generated from the dummy atoms of the envelope. The phases, which were generated with the P-domain envelope, were improved by the CCA method. In the CCA calculation, we used in-house Python and shell scripts together with *MAPROT* (Stein *et al.*, 1994 ▸) and programs from the *CCP*4 suite. The basic iteration process was the same as that for NCSA in §[Sec sec2]2, with averaging and solvent flattening in real space and Rayment weighting in reciprocal space. Solvent flattening was performed by filling zero values for densities in the solvent regions of each map.

In the CCA cycle, all reflections above 3.0 Å resolution were concurrently used from the first iteration, thus differing from the phase-extension method. From the MR result for the P3 data set, the P-domain envelope shows the trimeric NCS upon locating the crystallographic threefold symmetry. The trimeric NCS averaging was further included in the calculations. The number of cycles was 10 000, which sufficed for convergence (>200 cycles). The correlation-coefficient values were 0.617, 0.779 and 0.710 for P212121, C2D and C2S, respectively. After phase improvement by CCA, the C2S data were used to build the model, because its resolution was the highest (1.80 Å) at the time. *ARP*/*wARP* (Langer *et al.*, 2008 ▸) built 98% of the total residues automatically in one trial. We subsequently obtained new data to higher resolution (1.2 Å) from one *P*2_1_2_1_2_1_ crystal, and the structure was subjected to a final refinement with this high-resolution data set. The refinement was performed by *PHENIX* (Adams *et al.*, 2010 ▸) and *REFMAC*5 (Murshudov *et al.*, 2011 ▸). Table 1 ▸ presents the refinement statistics.

## 4. Discussion   

### 4.1. NCSA *ab initio* phasing   

The *ab initio* method with NCSA and phase extension has been successfully implemented to determine the structure of the *T* = 3 GNNV icosahedral VLP. The key to success was the similarity of the mask (or the envelope) to the target structure. Despite significant variations in the main chains of the CP between FHV and GNNV, the envelopes of these *T* = 3 capsids are somewhat similar to each other, especially in the spatial position of the P-domain at the quasi-threefold axis. Considering that the P-domain of FHV is much smaller than that of GNNV, the radius of the mask was typically increased to 13 Å to cover the entire structure of GNNV. Other trials using the large spherical-shell mask, which was cut by the bisector planes between neighbouring virus particles, were not successful in phasing. On inspection of this spherical-shell mask, we found that it was so small, covering only half of the P-domain, as to result in a negative effect on the phasing. Tests investigating the simulation trials using errorless amplitudes, *F*_simulated_, which were calculated from the FHV model positioned in the unit cell of the *T* = 3 GNNV-LP crystals, led to a map with a rough envelope that was uninterpretable in the first trial. In the second trial using a revised mask, which was made from the rough envelope, an interpretable map was obtained even with the mask cutting a small portion of the P-domain. This result is similar to that described in a previous report (Taka *et al.*, 2005 ▸). In another simulation trial using the spherical mask with the addition of a hemispherical mask at the P-domain position that covered the entire P-domain region, the map was finally obtained as a Babinet inversion, which yielded a solution in which the phases were incremented by π and in the production of negative electron density for the protein region (Tsao *et al.*, 1992 ▸; Plevka *et al.*, 2011 ▸). Taken together, the similarity of the mask to the target protein is highly sensitive to the *ab initio* phasing, but an ideal mask is not invariably required at the initial step.

### 4.2. Cross-crystal averaging phasing of the P-domain   

As in the CCA method, the first trial without the trimeric NCS failed to build a model automatically with *ARP*/*wARP*. The second trial, including the trimeric NCS with NCSA and CCA, which was addressed in §[Sec sec3]3, was successful. To increase the constraints of averaging by including the trimeric NCS, the phase sets were shifted further towards the correct solution, from which we could construct the complete model. As for the initial phase, the envelope cut out from the density of the *T* = 3 GNNV-LP served as the initial model or as the initial MR model to search for the translocation matrices. The envelope had only dummy C atoms placed at grid points of the density map with a constant level. Before we tried using the cut-out density, which was expected to have better phase information than the envelope, the phasing succeeded. In CCA phasing, we could state that only the information of the envelope of the P-domain was used.

### 4.3. Quantification of the phasing strength of the molecular averaging   

To quantify the strength of averaging in the sense of restriction of the phases, we introduce the free fraction (ff). ff is a simple indicator, which is easily calculated, while general to NCSA and CCA. When the copy number of NCS is 1, the ff value is identical to a fractional volume of protein molecules to be determined in the cell volume.

When the copy number of NCS is *n*, ff is expressed as
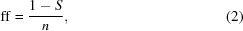
where *S* is the fraction of the solvent region. The solvent region is constrained to a constant value with solvent flattening. Furthermore, by the averaging restriction, the electron density of 1/*n* of the protein region can only be changed to reproduce the amplitude *F*_obs_ without constraint. Alternatively, it can be explained as that the densities of the 1 − 1/*n* protein fractional volume are restricted to the same densities as that of the 1/*n* protein volume.

We can thus expand the same concept for the CCA case,
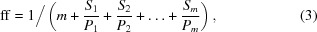
in which *S_k_* is the fraction of the solvent region of the *k*th crystal, *P_k_* is the protein fraction of the *k*th crystal and the equation *S*_*k*_ + *P*_*k*_ = 1 holds. A detailed derivation of (3) is shown in Appendix *A* [App appa]. In the case where *S*_*k*_ + *P*_*k*_ ≠ 1, for example where the solvent mask and the NCS mask are different; *P_k_* can be substituted by 1 − *S_k_*.

If we generalize the equation to *m* pieces of crystal, the *k*th crystal has *n_k_* NCS. We can write
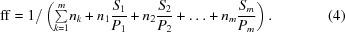



This ff value is another expression for the ratio of the number of data to the number of parameters (Appendix *B* [App appb]). The total data (amplitudes) with their phases can reproduce the densities over the whole volume of the crystal unit cell at corresponding grid spacing. When ff is equal to 0.5, which means that half of the densities are fixed and the other half can be varied as free parameters to reproduce the amplitudes, it is the same condition as when the missing phases amount to half of the total number of parameters for the construction of a Fourier synthesis map. This value is the largest value (0.5) to determine a unique solution of the electron-density map. In the case of ff > 0.5, the system is deemed to be ‘underdetermined’ or ‘overfitted’ and has no unique solution. Relative to these values of ff, the ‘constraint ratio’ has been discussed in Millane & Arnal (2015 ▸).

When *ab initio* phasing for the *T* = 3 GNNV-LP was successful, an FHV mask with a mask radius of 13 Å had a 0.71 fraction of the protein region; the copy number of NCS (nNCS) for averaging was 30. The ff value was hence 0.024 (= 0.71/30). We performed trial calculations varying the ff values by decreasing the copy number of NCS with the initial FHV mask and with the initial NCS matrix obtained from the results of self-rotation functions. The results are summarized in Table 2 ▸.

The quality of the map deteriorated with a smaller copy number of NCS, as expected. Notably, the *R* factor and CC values have a tendency to deteriorate with an increased number of NCSA or a decreased ff value because of the decreased number of degrees of freedom. Considering that the superior *R* factor and CC values are not invariably meant to help to obtain the correct phases, it is thus necessary to take these common phenomena into account in parameter searching when monitoring criteria such as *R* factor and CC. The phase differences from the refined model are presented in Table 2 ▸. When the map suffers from Babinet inversion, its phase differences deviate greatly, which is caused by the mixing of another phase set. Eliminating the case affected by Babinet inversion, the phase differences gradually improve with smaller ff values.

We also performed trial calculations varying the ff values by altering the mask volume. To increase or to decrease the mask volume, we extended or decreased the inner radius of the mask of FHV while the outer particle side of the mask was fixed. The mask with the inner radius extended to either 110 or 90 Å resulted in a non-interpretable density map. The trend in the variation of the ff value is difficult to obtain from simply varying the sizes of the mask volume. Not only a good coverage of the target molecule but also a suitable shape of the mask or envelope is critical for the success of *ab initio* phasing.

The phasing results of the truncated P-domain with various ff values are summarized in Table 3 ▸. The calculations in the upper rows are a successful case in terms of automatic model building with *ARP*/*wARP*, compared with those in the lower rows, which failed. The trimeric symmetries were included in the calculation of the successful upper case, but were excluded in the calculation of the failed lower case; the same mask was used in both calculations. The *R* factor and CC values seem to be random, and might be affected by the structural similarity of each molecule and the validities of their transfer matrices, although these values deviate less in the upper case. In both cases, phase extension was not utilized because of a lack of low-resolution (>30 Å) data. The phase difference (57.4°) from the refined model was smaller in the successful case, whereas the value of the phase difference (81.8°) for the failed case was large and did not decrease from the initial value. ff seems to act as an effective indicator in these phasing calculations.

The other factors in molecular averaging, such as the mask, transfer matrices and molecular equalities, as well as the data quality, must also be considered and evaluated. In the NCSA case, the mask roughly resembled the target envelope in shape and completely covered it. The transfer matrices and molecular isomorphism were expected to be satisfactory in the surface domains because of the nature of the icosahedral symmetry. The data quality, including the completeness of the very low resolution data, was essentially satisfactory. An ff value of ∼0.1 seems to be appropriate to separate successful (<0.1) and unsuccessful (>0.1) calculations in terms of map qualities for model building with NCSA (Table 2 ▸). In the CCA case, the mask from its own electron density was thought to be almost ideal. The CCA transfer matrices and the CCA isomorphism of molecules among the crystals were expected to be less satisfactory than those of the viral NCS. The data quality was satisfactory, but low-resolution (>30 Å) data were not measured. Coincidentally, an ff value of ∼0.1 was also found as a midpoint threshold to differentiate successful and failed calculations with CCA (0.038 and 0.113, respectively, in this work). From these analyses, we can estimate the ff values of both practical cases of NCSA and CCA, and suggest a new criterion of an ff value of 0.1 as a new rule of thumb to phase new structures of viruses with a single CP or proteins with highly redundant NCS or with several crystal forms.

## Supplementary Material

Supplementary Figure S1.. DOI: 10.1107/S2059798316007695/mh5197sup1.pdf

## Figures and Tables

**Table 1:** Statistics of data collection, phasing and refinement Values in parentheses are for the highest resolution shell.

Crystal (PDB code)	*T* = 3 GNNV-LP (4wiz)	Truncated P-domain				
		C2D	C2S	P212121 (4rfu)		P3
				Refinement	Phasing	
Data collection						
Wavelength (Å)	0.900	1.000	1.000	1.000		1.000
Temperature (K)	100	100	100	100		100
Space group	*C*2	*C*2	*C*2	*P*2_1_2_1_2_1_		*P*3
Unit-cell parameters						
*a* (Å)	477.4	149.2	88.7	64.9		260.8
*b* (Å)	422.7	85.4	83.8	82.5		260.8
*c* (Å)	337.9	82.5	58.9	87.7		250.5
α (°)	90.0	90.0	90.0	90.0		90.0
β (°)	134.0	117.0	106.0	90.0		90.0
γ (°)	90.0	90.0	90.0	90.0		120.0
Resolution (Å)	266.0–3.60 (3.66–3.60)	30.0–1.85 (1.92–1.85)	30.0–1.80 (1.86–1.80)	30.0–1.20 (1.24–1.20)	30.0–1.83 (1.90–1.83)	30.0–2.10 (2.18–2.10)
*R*_meas_ (%)	22.8 (98.8)	8.2 (55.2)	7.7 (46.2)	4.8 (47.7)	6.3 (45.0)	12.5 (50.9)
〈*I*/σ(*I*)〉	7.7 (1.5†)	17.7 (3.2)	18.9 (2.7)	31.3 (2.5)	32.1 (5.2)	14.7 (4.1)
Completeness (%)	99.4 (92.0)	99.3 (99.4)	99.7 (98.4)	99.2 (95.1)	99.8 (100)	100.0 (100.0)
Multiplicity	4.0 (2.7)	4.4 (4.3)	4.1 (3.6)	5.4 (2.7)	7.2 (7.3)	6.0 (6.0)
Phase extension (266–3.1 Å)‡						
Averaging *R* factor	0.276					
Correlation coefficient	0.885					
Refinement						
Resolution (Å)	266–3.60	30–1.85	30–1.80	30–1.20		30.0–2.10
*R*_work_ §/*R*_free_ ¶ (%)	25.5/29.7	16.9/19.3	16.8/19.8	17.5/18.1		20.6/24.5
No. of atoms						
Protein	203160	5480	2802	2739		2728
Ligand	90	58	26	31		—
Water	—	698	319	542		202
*B* factors (Å^2^)						
Protein	103.78	24.57	24.01	13.76		30.16
Ligand	38.14	38.89	40.72	22.76		—
Water	—	33.77	40.89	23.56		33.87
R.m.s. deviations						
Bond lengths (Å)	0.016	0.007	0.007	0.008		0.008
Bond angles (°)	1.767	1.055	1.066	1.044		1.144

† 〈*I*/σ(*I*)〉 in the outer shell is 1.5 (<2.0). We used this outer shell resolution because the high redundancy of NCS (= 30) is expected to work as the redundancy of the data. 〈*I*/σ(*I*)〉 falls below 2.0 in the 3.80–3.73 Å resolution shell.

‡ The resolution limit for the ‘final’ phase extension was extended to the outermost reflections while testing the map quality.

§ *R*_work_ = 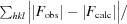 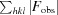, where *F*_obs_ and *F*_calc_ are the observed and calculated structure-factor amplitudes, respectively, of reflection *hkl*.

¶ *R*_free_ is as *R*_work_ but was calculated with 5% of randomly chosen reflections omitted from refinement.

**Table 2:** Map qualities depend on ff values by changing the number of NCS in the structure determination of *T =* 3 GNNV-LP

nNCS	ff	*R* factor	CC	Map quality	Phase difference (°)
30	0.024	0.254	0.882	Good	35.1
20	0.036	0.253	0.884	Better, inversion	88.3
10	0.071	0.241	0.892	Better	35.3
6	0.119	0.233	0.897	Main chain, inversion	87.3
5	0.142	0.231	0.902	Main chain, inversion	90.1
3	0.237	0.220	0.910	Uninterpretable	54.2
2	0.356	0.206	0.923	Main chain	67.2

**Table 3:** The outcome of model building depends on the ff values on changing the number of NCS and cross-crystal molecules in the structure determination of the P-domain

	ff	Crystal	*R* factor	CC	Phase difference (°)	Model building
(1 + 2 + 1) × 3 = 12 molecules in three crystals	0.038	P212121	0.408	0.617	57.4	Automatically built almost all in the first trial
		C2D	0.319	0.779		
		C2S	0.333	0.710		
(1 + 2 + 1) = 4 molecules in three crystals	0.113	P212121	0.496	0.392	81.8	Failed
		C2D	0.303	0.781		
		C2S	0.295	0.752		

## Appendix A Derivation of the equations for ff

Given the volume *p* of a protein molecule in an NCS unit and the solvent-region volume *s*, the density of the protein volume can be changed while the solvent volume is constrained to a fixed value. The ratio of the freely changing volume in the entire volume is written as

If the protein region is composed of *n* NCS molecules, only the density of one molecule can be free; the density for the other *n* − 1 molecules is constrained to the density of the first molecule. Therefore,

We rewrite this equation using the fraction of the solvent region *S*,

We expand the CCA case in the same way. Supposing that there are *m* crystals, which all have one molecule in NCS, and that *s_k_* and *p_k_* denote the volumes of the solvent and protein for the *k*th crystal, respectively, ff is written as

Furthermore,

and

in which *S_k_* and *P_k_* denote the fractions of the solvent and the protein regions, respectively, in the *k*th crystal.

Substituting (8) and (9) into (7), (3) is derived as

If we generalize the equation to *m* pieces of crystal, where the *k*th crystal has *n_k_* NCS, we substitute *n_k_* + *n_k_*(*S_k_*/*P_k_*) for 1 + (*S_k_*/*P_k_*). We rewrite the equation as

## Appendix B Comparison of the formulation of ff to phasing power

In early work by Arnold & Rossmann (1986 ▸) to quantify the phasing power with NCS, the square of *n* was used to replace the factor *n* as

(from equation 17 in Arnold & Rossmann, 1986 ▸), where *N* is the redundancy of NCS and *U*/*V* is equal to 1/(1 − *S*). *f* is the proportion of data near 1.0 and  is the error. Both quantities vary little, so that

whereas our phasing power *P*′ and ff have the relation

The solvent region and *n* − 1 of the protein region are equally counted as regions of constrained density. Our formulation is the result from the point of view of a ratio of data and parameters. In contrast, the formulation by Arnold and Rossmann is the result of an analogy to the effect of the multiple measurements on precision.
