# Supplementary material for: Ab initio phasing by molecular averaging in real space with new criteria: application to structure determination of a betanodavirus
Source: Acta Crystallogr D Struct Biol. 2016 Jun 23;72(Pt 7):830–40. doi: 10.1107/S2059798316007695 (PMC4932916; doi:10.1107/S2059798316007695)
Supplement: Supplementary file 1 [file d-72-00830-sup1.pdf]

STRUCTURAL
BIOLOGY

Volume 72 (2016)

Supporting information for article:

Ab initio phasing by molecular averaging in real space with new criteria: an application to structure determination of a betanodavirus

Masato Yoshimura, Nai-Chi Chen, Hong-Hsiang Guan, Phimonphan Chuankhayan, Chien-Chih Lin, Atsushi Nakagawa and Chun-Jung Chen

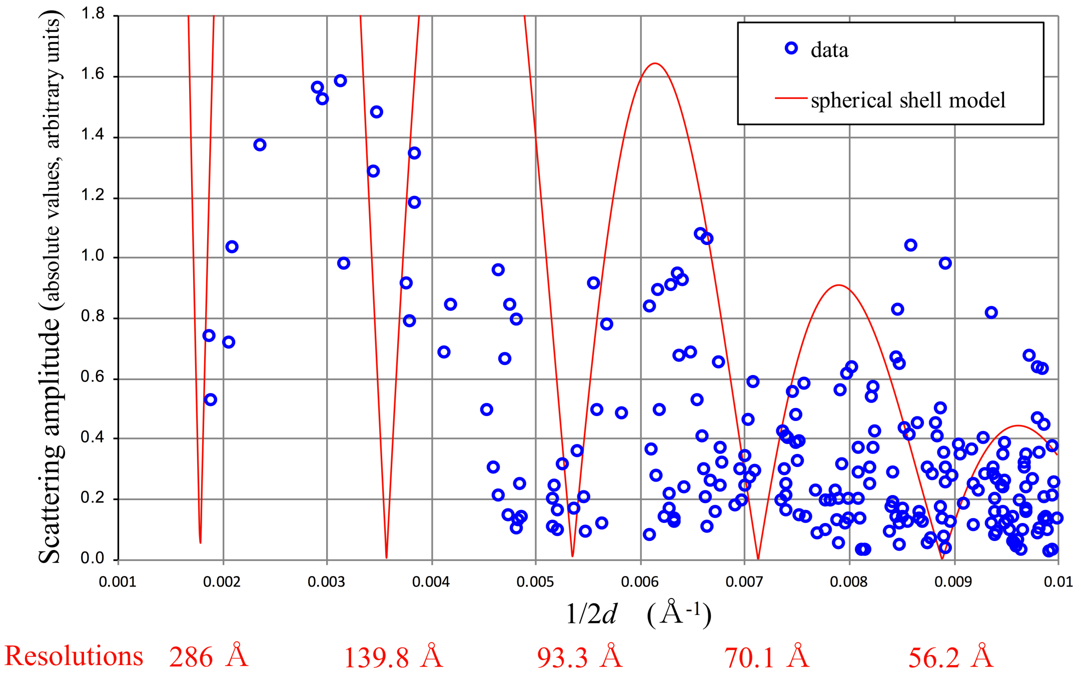

Figure S1 A comparison of the scattering amplitudes of the *T*=3 GNNV data and the absolute values of that of the spherical shell models at the low-resolution range (> 50 Å), of which the inner radius and the outer radius are 119 Å and 159 Å, respectively. The model amplitudes are not scaled to the data, but the scale is adjusted in order to emphasize the zero-crossing points (nodes) and the peaks. The resolutions at the nodes of the model amplitudes are shown at the bottom (red). Up to around the 4th node at resolution 70.1 Å, the model reproduces similar peaks and nodes relative to the data.
